# Lunar Surface Fault-Tolerant Soft-Landing Performance and Experiment for a Six-Legged Movable Repetitive Lander

**DOI:** 10.3390/s21175680

**Published:** 2021-08-24

**Authors:** Ke Yin, Songlin Zhou, Qiao Sun, Feng Gao

**Affiliations:** Key Laboratory of Mechanical System and Vibration, School of Mechanical Engineering, Shanghai Jiao Tong University, Shanghai 200240, China; jixie_yinke@sjtu.edu.cn (K.Y.); zhousonglin@sjtu.edu.cn (S.Z.); qiaosun1234@gmail.com (Q.S.)

**Keywords:** movable repetitive lander, fault-tolerant soft-landing, landing configuration, stability optimization

## Abstract

The cascading launch and cooperative work of lander and rover are the pivotal methods to achieve lunar zero-distance exploration. The separated design results in a heavy system mass that requires more launching costs and a limited exploration area that is restricted to the vicinity of the immovable lander. To solve this problem, we have designed a six-legged movable repetitive lander, called “HexaMRL”, which congenitally integrates the function of both the lander and rover. However, achieving a buffered landing after a failure of the integrated drive units (IDUs) in the harsh lunar environment is a great challenge. In this paper, we systematically analyze the fault-tolerant capacity of all possible landing configurations in which the number of remaining normal legs is more than two and design the landing algorithm to finish a fault-tolerant soft-landing for the stable configuration. A quasi-incentre stability optimization method is further proposed to increase the stability margin during supporting operations after landing. To verify the fault-tolerant landing performance on the moon, a series of experiments, including five-legged, four-legged and three-legged soft-landings with a vertical landing velocity of −1.9 m/s and a payload of 140 kg, are successfully carried out on a 5-DoF lunar gravity ground-testing platform. The HexaMRL with fault-tolerant landing capacity will greatly promote the development of a next-generation lunar prober.

## 1. Introduction

The moon is the hub and bridge between mankind and the universe, while lunar exploration is the premise and basis of deep space exploration. Nowadays, the separated design of an immovable lander and rover is still the core method of zero-distance exploration on the moon. Many countries have made world-renowned achievements such as the Soviet/Russian Luna-9 [1], the first lander to achieve lunar soft-landing, which absorbs impact energy using four airbags; American Surveyor-1 [2], the first legged lander to reach the lunar surface, which uses three three-branch buffered legs filled with aluminum honeycomb material, providing technical support for Apollo program [3]; Chinese Chang’e 4 [4] reaches the far side of the moon first, which utilizes four similar buffer legs. Notably, all these landers are immovable and are designed to help the rover finish landing, so their exploration capacity is restricted to around the fixed landing site. Two kinds of rover are applied to expand the exploration range, one is a manned lunar rover, like LRV [5], which can carry up to two astronauts, another is the unmanned wheeled rover, like Yutu 1 & 2 [4,6], which can maneuver quickly with scientific instruments. However, the separated design of the lander and rover creates a heavy and complex prober system. The rover only executes exploration in a circle district with the lander as the center and the safety distance as the radius.

Thanks to excellent traversing performance on irregular terrain [7,8], legged robots are promising to accomplish lunar exploration compared with a wheeled rover. On the one hand, the leg form is utilized in most existing landers, though they lack mobility on the lunar surface. On the other hand, legged robots are well designed in many fields such as running robots, Bigdog [9], Cheetah-3 [10] and Anymal [11,12]; underwater robots, Crabster [13,14]; heavy-duty robots, Octopus [15]; and exploration robots, Athlete [16], Spaceclimber [17] and Spacebok [18,19]. Furthermore, to combine the excellent speed performance for wheeled robots on even terrain and a great adaptive capacity for legged robots on irregular terrain, the wheel-legged robot [20] adopted a hierarchical framework to control wheel and leg motions; this has drawn a lot of researcher attention. Nevertheless, the current legged or wheel-legged robots are difficult to directly apply to lunar exploration. The hydraulic actuator is widely employed in HyQ2Max [21] or Bigdog [9] to obtain high explosive torque, which is infeasible for extraterrestrial exploration. The leg layout of a running robot cannot withstand the landing impact in all directions. The buffer capacity of the current robot is relatively weak, so the engine nozzle under the lander body will be easily damaged for colliding with the ground.

We have designed a six-legged movable repetitive lander “HexaMRL” in previous work to integrate the function of both lander and rover. IDUs are used to simulate the dynamic characters of an active dissipative system of spring and dampener by impedance control to achieve a buffered landing and protect the leg structure, different from the irreversible deformation of aluminum honeycomb material after landing [22,23,24]. Hence, the lander can still execute locomotion as a rover. After exploration at a current landing site, it can fly to the next landing site using the engine and repetitively perform buffered landing tasks. This new exploration mode will significantly increase the utilization rate of an individual prober on the moon and greatly extend the exploration district. However, the repetitive work mode has higher requirements for the quality and fault-tolerant landing capacity of the lander.

In the harsh lunar environment (i.e., intense radiation, large temperature difference and ultravacuum), it is hard to repair with the remote operation if some failures occur on the IDUs. Fault-tolerant control (FTC) for the robot has attracted great attention all over the world and is pivotal for the prober to execute exploration tasks. Nowadays, FTC is generally achieved by the following three methods. Firstly, multiple drives are used in the active joints; for example, Zhang et al. [25] employ dual-input/single-output (DISO) to drive the servo press machine. Secondly, the parallel robot could use a redundant drive [26] to eliminate singularities in the workspace. The third one is to increase the DoF of robot end-effector, such as the Canadian space station’s remote manipulator system (SSRMS) [27], which adopted seven series joints to improve the workspace. However, the above FTC relies on more drives or more complex mechanisms, which will increase the system mass and complexity that are difficult for the lander to accept. Furthermore, fault-tolerant landing is not generally considered in current landers because most of the existing landers are three-legged or four-legged, which constructively lack fault-tolerant landing capacity when one leg fails. For a three-legged lander like Surveyor-1, the remaining two legs cannot support the lander. As for four-legged landers like Apollo 11 [3] or Chang’e 3, 4, & 5 [4,6,28], the center of mass of the lander will move to the side of the supporting triangle constructed by the remaining three legs, leading to a failed buffer landing on the uneven lunar surface. Therefore, hard strict standards are required for the manufacture and control of such landers and would be abandoned if any failures occur. 

The six-legged design in HexaMRL makes the fault-tolerant soft-landing feasible without any supplement of drivers or mechanisms. In this paper, we have systematically studied the fault-tolerant soft-landing performance on the moon for HexaMRL. Firstly, we analyze the classification and stability of the landing configuration and establish the relationship between fault number and landing configuration by the synthesis equation. Secondly, regarding stable configuration, the corresponding fault-tolerant landing algorithms are designed to achieve a buffered landing, and a quasi-incentre stability optimization method is further proposed to increase the stability margin during supported operations. Thirdly, to verify the fault-tolerant landing on the moon, a series of experiments including five-legged, four-legged and three-legged soft-landing with a vertical landing velocity of −1.9 m/s and a payload of 140 kg are successfully carried out on a 5-DoF lunar gravity ground testing platform. 

The rest of the paper is organized as follows. Section 2 introduces the lander system. The landing configuration is analyzed in Section 3. Section 4 and Section 5 design the fault-tolerant algorithm and optimize the stability margin in supporting the operations, respectively. Section 6 clarifies the fault-tolerant landing experiments, and Section 7 discusses the experiment results. The last section is the conclusion and its expansion.

## 2. Lander System

The HexaMRL is composed of six identical legs that adopt a hybrid mechanism actuated by three IDUs. During buffer landing, each IDU imitates the dynamic characters of both spring and damper. If some errors occur in the IDUs of the leg under the harsh lunar environment, the leg residual mobility capacities are a great variant in different fault combinations. 

### 2.1. HexaMRL

**Figure 1:**
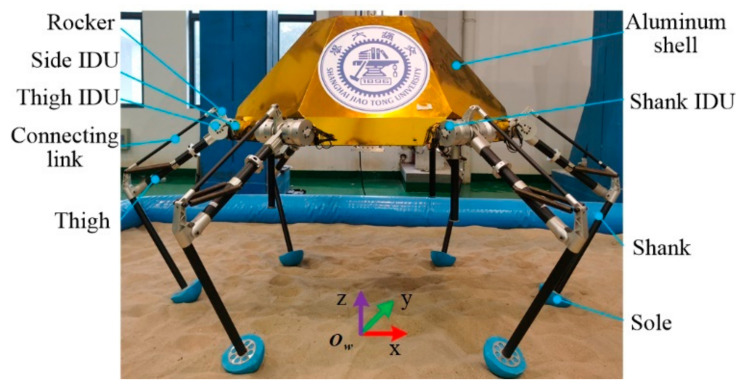
The six-legged movable and repetitive lander (HexaMRL).

As shown in Figure 1 the HexaMRL is designed to execute repetitive soft-landing and roving for lunar exploration. Its size is about 1.35 m long, 0.94 m wide and 0.75 m high; it weighs 60 kg, including a 20 kg aluminum shell. The robot consists of a body and six identical legs. Each leg is composed of side IDU, thigh IDU, shank IDU, thigh, rocker, connecting link, sole and shank. All legs are connected to the body that carries the controller, inertial measurement unit (IMU), power system, sensing system, payloads, etc. 

**Figure 2:**
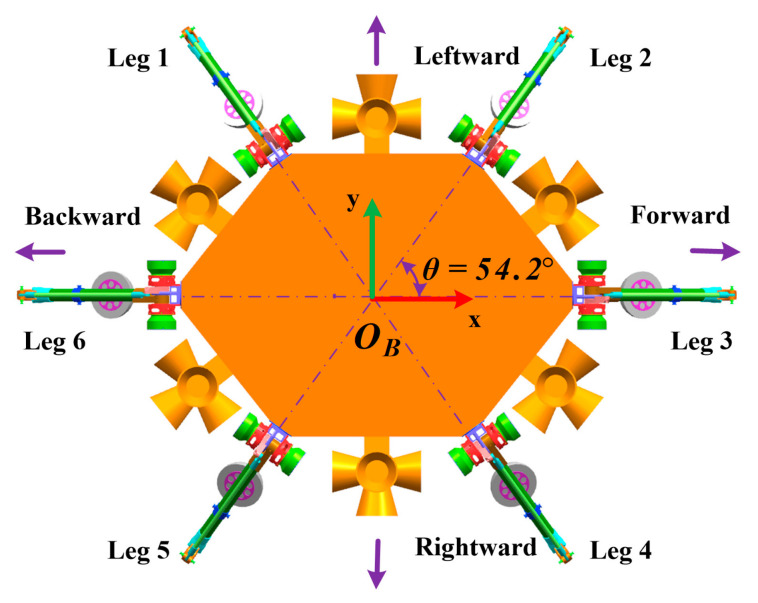
The leg distribution of HexaMRL.

The leg distribution design needs to meet landing and roving functions simultaneously. On the one hand, to withstand the impact force uniformly during buffer landing, all legs are arranged as an equilateral polygon like a regular triangle in Surveyor-1 [2] or Square in Apollo program [3] and Chang’e series [4,6,28]. As for the six-legged lander, the angular interval between adjacent legs should be 60°. On the other hand, to achieve quick locomotion, all legs will be laid out in a slender shape as in animals like the cheetah, lion or goat, like the hexapod robot RHex [29] or TUM-walking machine [30]. Eventually, as illustrated in Figure 2, the angle between leg 2 and leg 3 is designed to be 54.2°. Leg 1 (or 2) and leg 5 (or 4) are symmetric about axis-*x*, while leg 1 (or 5) and leg 2 (or 4) are symmetric about axis-*y*.

### 2.2. Leg Mechanism

**Figure 3:**
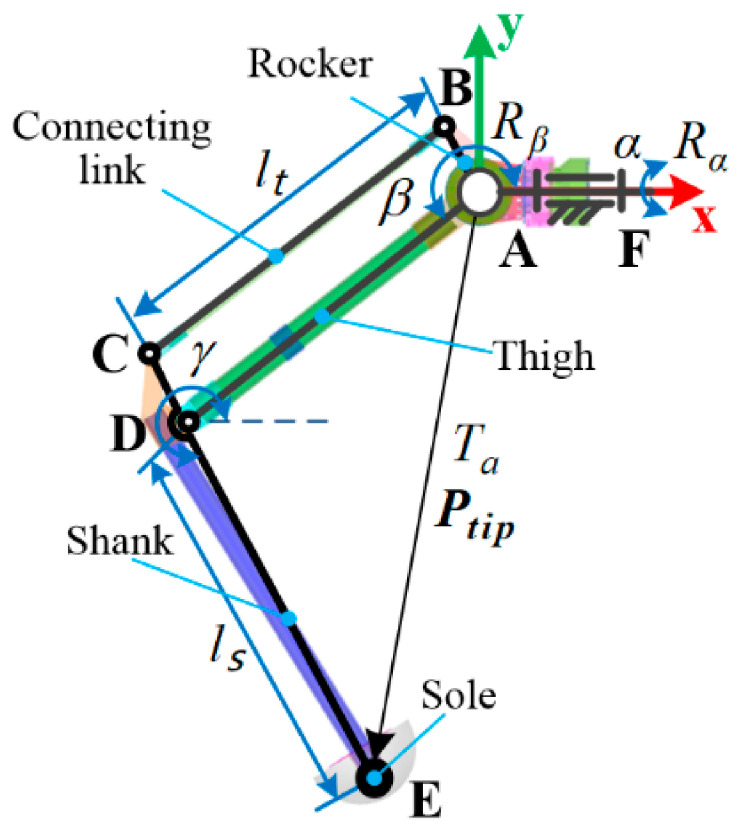
The leg mechanism.

The leg mechanism is presented in Figure 3. It has three active DoFs that are actuated by side IDU, thigh IDU and shank IDU to control the mobility of the rocker, thigh and shank, respectively. Let $q={\left[\begin{array}{ccc} \alpha & \beta & \gamma \end{array}\right]}^{T}$ denote the generalized coordinate vector where α, β and γ are the joint angles of the side, thigh and shank, respectively. The tiptoe position $P_{tip}$ in the leg coordinate frame can be obtained as follows:

(1) $$P_{tip}=\left[\begin{array}{c} l_{t}cos\beta +l_{s}cos\gamma \\ cos\alpha \left(l_{t}sin\beta +l_{s}sin\gamma \right)\\ sin\alpha \left(l_{t}sin\beta +l_{s}sin\gamma \right) \end{array}\right]$$

where $l_{t}$ and $l_{s}$ are the length of the thigh and shank, separately.

The IDU consists of an encoder, servo motor, torque sensor, harmonic reducer, shell, coupler, bearing, etc. During buffer landing, each IDU imitates the dynamic characters of an active torsion spring and an active torsion damper by the impedance control method, whose control rule can be written as follows:

(2) $$\tau_{act}-\tau_{des}=K_{a}\left(\varphi_{des}-\varphi_{act}\right)+B_{a}\left({\dot{\varphi }}_{des}-{\dot{\varphi }}_{act}\right)$$

where $\tau_{act}$ and $\tau_{des}$ are the actual torque and desired torque of IDU, $K_{a}$ and $B_{a}$ are the coefficients of stiffness and damping of active compliance, $\varphi_{des}$ and $\varphi_{act}$ are the desired angle and actual angle of the active joint, ${\dot{\varphi }}_{des}$ and ${\dot{\varphi }}_{act}$ are corresponding angular velocity.

### 2.3. Leg Residual Capacity

**Figure 4:**
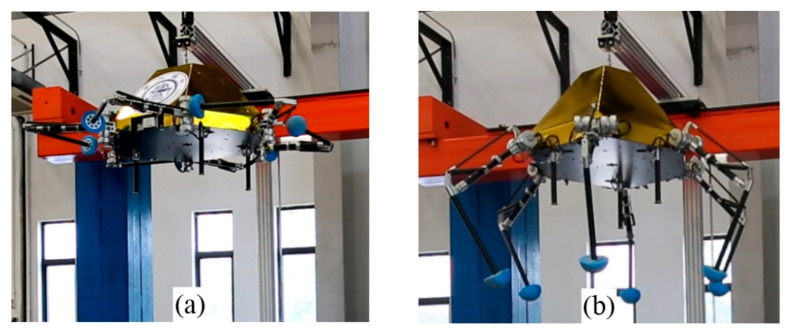
Leg function. (**a**) folding; (**b**) deploying.

In order to reduce the rocket size, all legs need folding, as in Figure 4a, for compact volume when launched from Earth. Before the buffer landing on the moon, all legs need to deploy from the folded state, as in Figure 4b, to obtain better supporting stability and a larger buffer stroke. Since the mobility demand before and after the deploying task is not the same, the fault-tolerant landing capacity is significantly different as well. 

**Figure 5:**
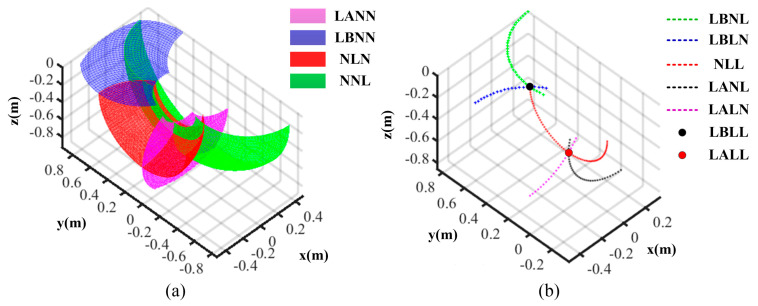
The leg residual workspace. (**a**) One fault; (**b**) two or three faults.

Here, because the capacities of fault-tolerant are different, we use LB and LA to distinguish the time of fault occurrence on side IDU before and after deploying operation. On the contrary, as for the thigh or shank IDU, distinguishing separate failure times is unnecessary due to them having the same capacities. The normal IDU of the thigh or shank is denoted by N, while the failed IDU is represented by L. When some IDUs are failed, the residual workspaces are illustrated in Figure 5. There are four cases when one IDU fails. Firstly, if the side IDU fails after deploying, and the other two IDUs are normal, this case can be denoted by LANN. Its residual workspace is the purple vertical plane, and the mobility character is expressed as $G_{F}^{II}\left(0,\;R_{\beta },0;T_{a},0,0\right)$ by $G_{f}$ theory [31]. Where $R_{\beta }$ and $T_{a}$ are the swing movement and the stretching/shrinking movement in the leg sagittal plane. Secondly, if the side IDU fails before deploying and the other two IDUs are normal, denoted by LBNN, the residual workspace is the blue horizontal plane, and the mobility character is expressed as $G_{F}^{II}\left(0,\;R_{\beta },0;T_{a},0,0\right)$. Thirdly, if the thigh IDU fails, denoted by NLN, the residual workspace is the red surface, and the mobility character is expressed as $G_{F}^{II}\left(R_{\alpha },\;R_{\beta },0;0,0,0\right)$ where $R_{\alpha }$ is the abduction/adduction movement. Lastly, if the shank IDU fails, denoted by NNL, the residual workspace is the green surface, and the mobility character is expressed as $G_{F}^{II}\left(R_{\alpha },\;R_{\beta },0;0,0,0\right)$. There are five cases if two IDUs fail, separately denoted by LBNL, LBLN, NLL, LANL and LALN. Their residual workspaces are the dotted curves of green, blue, red, black and purple; their corresponding mobility characters are expressed as $G_{F}^{II}\left(0,\;R_{\beta },0;0,0,0\right)$ or $G_{F}^{II}\left(R_{\alpha },\;0,0;0,0,0\right)$. There are two cases if all three IDUs fail, denoted by LBLL and LALL; their residual workspaces are a fixed point of black and red while the mobility characters are $G_{F}^{I}\left(0,\;0,0;0,0,0\right)$.

Noticeably, the lander must possess the up/down movement character during the buffer landing period. Therefore, only the fault case LANN satisfies the mobility demand and has the fault-tolerant landing capacity, further symbolized as $E1_{1}$. The other three cases with one fault don’t have fault-tolerant landing capacity and are symbolized as $E1_{2}$, uniformly. All cases with two or three faults cannot finish fault-tolerant landing, so the fault time before or after deploying operation will not be distinguished, and the symbol LA or LB will be replaced by L. Finally, the two faulted IDUs cases are denoted as E2, while the three faulted IDUs case is written as E3. The detailed fault-tolerant capacity of the leg is shown in Table 1.

## 3. Landing Configuration Analysis

When more than one IDU fails, different legs may be involved, and their spatial distribution will affect the landing performance. The landing performance will be determined by the configuration that consists of the supporting polygon of the foothold of the remaining normal legs and the position of the lander’s center of mass. The stabilities in landing configurations are different, and we systematically assess this by the dimensionless index SAI. Then, we establish a synthesis equation to deduce all possible configurations under a certain number of failed IDUs.

### 3.1. Classification

Specifically, we need to consider the equivalent leg when the landing configurations are analyzed. As shown in Figure 2, all legs have different effects on landing performance because their interval angles are not equal to 60°. However, legs 1, 2, 4 and 5 are a group of equivalent legs while legs 3 and 6 are the other group, and the influence of equivalent legs on landing performance is the same.

Noticeably, there are three basic properties for landing configuration. (1) The landing performance is different if the landing configuration is different. (2) The configuration is the same if one equivalent leg fails, e.g., leg 1 or 2 failed. (3) If configuration 1 coincides with configuration 2 after rotation and symmetry operations, the two configurations are considered the same, e.g., legs 1 and 3 or legs 3 and 6.

**Figure 6:**
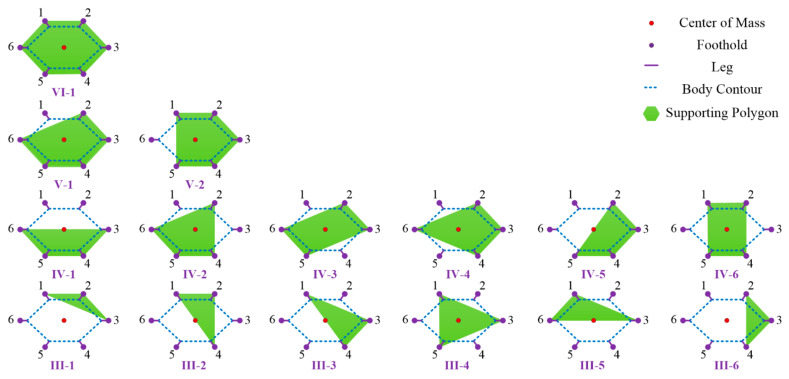
Classification of landing configuration.

The classification of landing configuration is shown in Figure 6. The red point denotes the center of mass, while the purple points express the footholds. The solid purple lines illustrate the leg position, while the blue dotted lines show the body contour. The supporting polygon constructed by the footholds of remaining normal legs is denoted by the green plane. The relationship between the normal supporting leg and landing configuration is illustrated in Table 2. As for six-legged landing, there is only one configuration ($C_{6}^{6}=1$) called VI-1. As for five-legged landing, there are six configurations ($C_{6}^{5}=6$) that are further divided into two groups: V-1 includes supporting leg 2-3-4-5-6, 1-3-4-5-6, 1-2-3-5-6 and 1-2-3-4-6; V-2 consists of supporting leg 1-2-4-5-6 and 1-2-3-4-5. As for four-legged landing, there are fifteen configurations ($C_{6}^{4}=15$) that are detailly classified into six groups: IV-1 includes supporting leg 3-4-5-6 and 1-2-3-6; IV-2 includes supporting leg 2-4-5-6, 1-3-4-5, 1-2-4-6 and 1-2-3-5; IV-3 includes supporting leg 2-3-5-6 and 1-3-4-6; IV-4 includes supporting leg 2-3-4-6 and 1-3-5-6; IV-5 includes supporting leg 2-3-4-5, 1-4-5-6 and 1-2-5-6; IV-6 includes supporting leg 1-2-4-5. As for three-legged landing, there are twenty configurations ($C_{6}^{3}=20$) categorized in six groups: III-1 includes supporting leg 1-2-3, 1-2-6, 3-4-5 and 4-5-6; III-2 includes supporting leg 1-2-4, 1-2-5, 1-4-5 and 2-4-5; III-3 includes supporting leg 1-3-4, 1-4-6, 2-3-5 and 2-5-6; III-4 includes supporting leg 1-3-5 and 2-4-6; III-5 includes supporting leg 1-3-6, 2-3-6, 3-4-6 and 3-5-6; III-6 includes supporting leg 2-3-4 and 1-5-6. Here, the analysis of configuration consisting of one or two legs is omitted because the lander cannot finish a fault-tolerant landing. 

### 3.2. Stability

The influence of different configurations on landing stability is determined by the shortest distance from the ground projection point of the center of mass to each side of the supporting polygon (d) and the area of the supporting polygon (S). The dimensionless index SAI is proposed to evaluate the stability of each configuration and is written as follows:

(3) $$SAI=\frac{d}{d_{VI}}*\frac{S}{S_{VI}}$$

where $d_{VI}$ and $S_{VI}$ are the values of d and S in normal six-legged landing configuration VI, respectively. If the center of mass is within the supporting polygon, then d>0. On the contrary, d<0. 

Eventually, the SAI value in each configuration can be calculated because the legs are deployed to the predetermined position from the folded status for the buffer landing. According to the SAI, the configuration stabilities will be divided into three statuses: stable, critical stable and unstable, and can be written as follows:

(4) $$SS=\left\{\begin{array}{c} Stable\;\left(S\right),\;\;\;if\;SAI>0;\\ Critical\;Stable\;\left(CS\right),\;\;\;if\;SAI=0;\\ Unstable\;\left(US\right),\;\;\;if\;SAI<0. \end{array}\right.$$

As seen in Table 3, the stable configurations have eight cases: VI-1, V-1, V-2, IV-2, IV-3, IV-4, IV-6 and III-4, in which the robot can execute fault-tolerant landing. The critical stable configurations have five cases: IV-1, IV-5, III-2, III-3 and III-5. In these cases, the robot can only perform soft-landing if the landing site is absolutely even. The unstable configurations include III-1 and III-6. Owing to the irregular lunar surface, the lander cannot finish soft-landing under both critical stable and unstable configurations.

### 3.3. Relationship between Fault Number and Configuration

When the number of faulted IDUs is $N_{m}$, all the possible configurations will be concluded by the following equation:

(5) $$\left\{\begin{array}{c} \overset{3}{\underset{i=1}{\sum }}N_{i}\cdot i=N_{m}\\ \overset{3}{\underset{i=1}{\sum }}N_{i}=N_{L} \end{array}\right.$$

(6) $$s.t.\;\left\{\begin{array}{c} 0\leq N_{L}\leq min\left\{6,\;N_{m}\right\}\\ 0\leq N_{m}\leq 18\\ 0\leq N_{i}\leq 6,\;i=1,\;2,\;3 \end{array}\right.$$

where $N_{i}$ is the number of legs with i faulted IDUs, $N_{L}$ is the total number of failed legs. The solve set $\left\{\begin{array}{ccc} N_{1}, & N_{2}, & N_{3} \end{array}\right\}$ illustrates the faults distribution. Considering the fault-tolerant capacity of a single leg in Table 1, we can get the possible configuration details. When $N_{m}$ is 1, the solve set is $\left\{\begin{array}{ccc} 1, & 0, & 0 \end{array}\right\}$. The landing configuration is VI if the failed leg is $E1_{1}$. On the contrary, the landing configuration is V if the failed leg is $E1_{2}$. Similarly, when $N_{m}$ is 2, there are two solve sets. Different fault leg groups will result in VI, V, or IV configuration. Table 4 lists all possible landing configurations when the number of faulted IDUs is less than seven. Lastly, we can get landing configuration type II by substituting the number of failed legs into Table 2. If the faulted number is more than six, the combination results can be obtained by the above Equations (5) and (6), similarly.

## 4. Fault-Tolerant Landing Algorithms

During the buffer landing period, the force and torque equations of the lander can be written as:

(7) $$\left\{\begin{array}{c} F_{ez}=m_{b}g+\overset{N}{\underset{i=1}{\sum }}F_{giz}\\ \tau_{exy}=\overset{N}{\underset{i=1}{\sum }}\left(r_{c}+r_{bfi}\right)\times F_{giz} \end{array}\right.$$

where $m_{b}$ is the mass of the lander, g is the gravity acceleration on the moon, N is the number of normal supporting legs, $F_{giz}$ is the vertical supporting force of the i-th leg, $r_{c}$ is the vector from the center of mass to the origin $O_{b}$ of the body coordinate frame, $r_{bfi}$ is the vector from $O_{b}$ to the i-th foothold, $F_{giz}={\left[\begin{array}{ccc} 0 & 0 & F_{giz} \end{array}\right]}^{T}$ is the vector form of $F_{giz}$, $F_{ez}$ and $\tau_{exy}={\left[\begin{array}{cc} \tau_{ex} & \tau_{ey} \end{array}\right]}^{T}$ are dividedly the resultant force and torque.

By adjusting the leg force $F_{giz}$, we can control the stable body states. After landing, we desire a constant body height and a horizontal body plane. The $F_{ez}$ will be set to zero and the $\tau_{exy}$ will be employed to control the angle deviation of pitch and roll. The PID controller will generate the adjusted torque in real-time. Considering the desired stable values of angle and angular velocity are zero, the $\tau_{exy}$ will be calculated from the following equation:

(8) $$\left\{\begin{array}{c} \tau_{ex}=-k_{px}\theta_{ra}-k_{dx}{\dot{\theta }}_{ra}-k_{ix}\int \theta_{ra}dt\\ \tau_{ey}=-k_{py}\theta_{pa}-k_{dy}{\dot{\theta }}_{pa}-k_{iy}\int \theta_{pa}dt \end{array}\right.$$

where $k_{px}$, $k_{dx}$, $k_{ix}$, $k_{py}$, $k_{dy}$ and $k_{iy}$ are the proportional, integral and derivative gains in the x-axis and y-axis, respectively. $\theta_{ra}$ and $\theta_{pa}$ are the actual angles of roll and pitch. ${\dot{\theta }}_{ra}$ and ${\dot{\theta }}_{pa}$ are the corresponding velocities.

### 4.1. VI Configuration

In six-legged normal soft-landing, the force/torque balance equations can be written as follows in matrix form by substituting Equation (8) and $F_{ez}=0$ into Equation (7).

(9) $$\left[\begin{array}{cccccc} 1 & 1 & 1 & 1 & 1 & 1\\ r_{cy}+r_{bf1y} & r_{cy}+r_{bf2y} & r_{cy}+r_{bf3y} & r_{cy}+r_{bf4y} & r_{cy}+r_{bf5y} & r_{cy}+r_{bf6y}\\ r_{cx}+r_{bf1x} & r_{cx}+r_{bf2x} & r_{cx}+r_{bf3x} & r_{cx}+r_{bf4x} & r_{cx}+r_{bf5x} & r_{cx}+r_{bf6x} \end{array}\right]\cdot \left[\begin{array}{c} F_{g1z}\\ F_{g2z}\\ F_{g3z}\\ F_{g4z}\\ F_{g5z}\\ F_{g6z} \end{array}\right]=\left[\begin{array}{c} -m_{b}g\\ \tau_{ex}\\ \tau_{ey} \end{array}\right]$$

**Figure 7:**
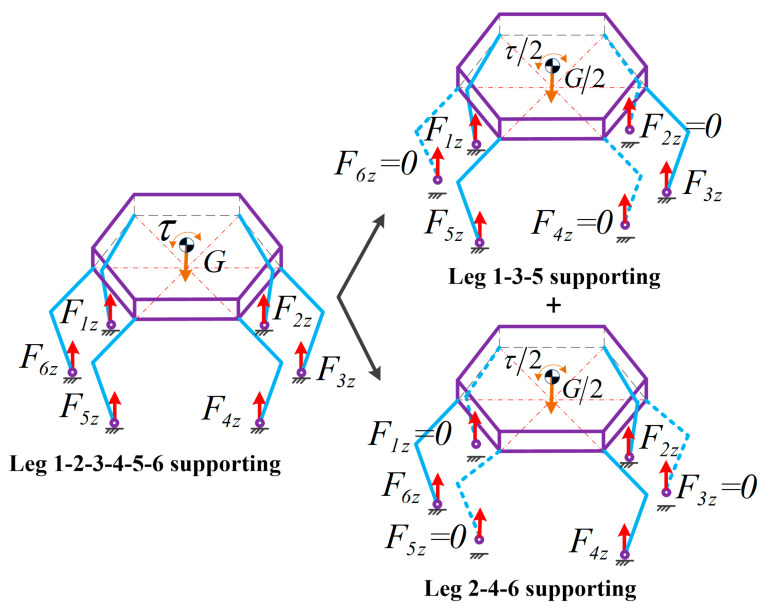
Virtual three-legged supporting method (VTLSM).

However, Equation (9) is an indeterminate equation group and has numerous solutions. It is time-consuming to solve the generalized inverse matrix in a real-time system. Here, a virtual three-legged supporting method (VTLSM) is proposed to allocate the adjusted force $F_{giz}$ quickly. As illustrated in Figure 7, we divide the six supporting legs into two groups: leg 1-3-5 supporting (group A) and leg 2-4-6 supporting (group B). During buffer landing, the legs of each group provide half the adjusted force and torque. Then Equation (9) can be rewritten as follows:

(10) $$\left[\begin{array}{ccc} 1 & 1 & 1\\ r_{cy}+r_{bfky} & r_{cy}+r_{bfmy} & r_{cy}+r_{bfny}\\ r_{cx}+r_{bfkx} & r_{cx}+r_{bfmx} & r_{cx}+r_{bfnx} \end{array}\right]\cdot \left[\begin{array}{c} F_{gkz}^{kmn}\\ F_{gmz}^{kmn}\\ F_{gnz}^{kmn} \end{array}\right]=\frac{1}{2}\left[\begin{array}{c} -m_{b}g\\ \tau_{ex}\\ \tau_{ey} \end{array}\right]$$

where k, m and n are the number of supporting legs in each group. Particularly, k=1, m=3 and n=5 in group A while k=2, m=4 and n=6 in group B. Lastly, the indeterminate Equation (9) is transformed into a two determinate Equation (10) that will generate the adjusted foot force $F_{giz}$ easily in real-time by solving the inverse matrix of a 3 × 3 matrix.

The desired torque of the joint impedance controller in Equation (2) comes from the following equation:

(11) $$\tau_{des}=J{\left(q\right)}_{f}^{-1}F_{g}$$

where $F_{g}={\left[\begin{array}{ccc} 0 & 0 & F_{gz} \end{array}\right]}^{T}$ is the vector form of adjusted leg force and $J{\left(q\right)}_{f}$ is the force Jacobian matrix.

### 4.2. V Configuration

As for five-legged landing, the 3 × 6 coefficient matrix in Equation (9) will be transformed into a 3 × 5 matrix that is still indeterminate. Here we chose configuration V-2 to illustrate the allocated process of leg forces. The force/torque balance equations are written as:

(12) $$\left[\begin{array}{ccccc} 1 & 1 & 1 & 1 & 1\\ r_{cy}+r_{bf1y} & r_{cy}+r_{bf2y} & r_{cy}+r_{bf3y} & r_{cy}+r_{bf4y} & r_{cy}+r_{bf5y}\\ r_{cx}+r_{bf1x} & r_{cx}+r_{bf2x} & r_{cx}+r_{bf3x} & r_{cx}+r_{bf4x} & r_{cx}+r_{bf5x} \end{array}\right]\cdot \left[\begin{array}{c} F_{g1z}\\ F_{g2z}\\ F_{g3z}\\ F_{g4z}\\ F_{g5z} \end{array}\right]=\left[\begin{array}{c} -m_{b}g\\ \tau_{ex}\\ \tau_{ey} \end{array}\right]$$

The VTLSM will be again used to quickly solve the foot force. There are ten virtual triangles ($C_{5}^{3}=10$) constructed by any three supporting legs. They are divided into two cases according to the ground projected position of the center of mass of the lander. If this point is inside the virtual supporting triangles, these triangles are valid, and the number of them is $N_{v}$. On the contrary, if the others are invalid and their number is $N_{i}$. The supporting legs of the valid triangle are leg 1-2-4, leg 1-2-5, leg 1-3-4, leg 1-4-5, leg 2-3-5 and leg 2-4-5, while the ones of the invalid triangle are leg 1-2-3, leg 2-3-4 and leg 3-4-5. Then the $N_{v}$ is seven and $N_{i}$ is three. Meanwhile, the Equation (10) is modified as follows:

(13) $$\left[\begin{array}{ccc} 1 & 1 & 1\\ r_{cy}+r_{bfky} & r_{cy}+r_{bfmy} & r_{cy}+r_{bfny}\\ r_{cx}+r_{bfkx} & r_{cx}+r_{bfmx} & r_{cx}+r_{bfnx} \end{array}\right]\left[\begin{array}{c} F_{gkz}^{kmn}\\ F_{gmz}^{kmn}\\ F_{gnz}^{kmn} \end{array}\right]=\frac{1}{N_{v}}\left[\begin{array}{c} -m_{b}g\\ \tau_{ex}\\ \tau_{ey} \end{array}\right]$$

Noticeably, the i-th supporting leg provides adjusted force in multiple valid triangles, so we will obtain the eventual foot force by the following sum operation:

(14) $$\left\{ \begin{array}{l} F_{g1z}=F_{g1z}^{124}+F_{g1z}^{125}+F_{g1z}^{134}+F_{g1z}^{135}+F_{g1z}^{145}\\ F_{g2z}=F_{g2z}^{124}+F_{g2z}^{125}+F_{g2z}^{235}+F_{g2z}^{245}\\ F_{g3z}=F_{g3z}^{134}+F_{g3z}^{135}+F_{g3z}^{235}\\ F_{g4z}=F_{g4z}^{124}+F_{g4z}^{134}+F_{g4z}^{145}+F_{g4z}^{245}\\ F_{g5z}=F_{g5z}^{125}+F_{g5z}^{135}+F_{g5z}^{145}+F_{g5z}^{235}+F_{g5z}^{245} \end{array}\right.$$

In configuration V-1, we will just change the leg number to 2, 3, 4, 5 and 6. Then the adjusted force can be found by the similar Equations (13) and (14). The detailed classification of the virtual supporting triangle is listed in Table 5.

### 4.3. IV and III Configurations

As for four-legged landing, the 3 × 6 coefficient matrix in Equation (9) will be transformed into a 3 × 4 matrix. For example, in IV-2 configuration, the force/torque balance equations are written as:

(15) $$\left[\begin{array}{cccc} 1 & 1 & 1 & 1\\ r_{cy}+r_{bf2y} & r_{cy}+r_{bf4y} & r_{cy}+r_{bf5y} & r_{cy}+r_{bf6y}\\ r_{cx}+r_{bf2x} & r_{cx}+r_{bf4x} & r_{cx}+r_{bf5x} & r_{cx}+r_{bf6x} \end{array}\right]\cdot \left[\begin{array}{c} F_{g2z}\\ F_{g4z}\\ F_{g5z}\\ F_{g6z} \end{array}\right]=\left[\begin{array}{c} -m_{b}g\\ \tau_{ex}\\ \tau_{ey} \end{array}\right]$$

Similarly, we utilize the VTLSM to calculate the adjusted leg force in real-time. The classification of the virtual supporting triangle is listed in Table 6.

As for three-legged landing, the 3 × 6 coefficient matrix in Equation (9) will be transformed into a 3 × 3 matrix. For configuration III-4, the equation is written as follows:

(16) $$\left[\begin{array}{ccc} 1 & 1 & 1\\ r_{cy}+r_{bf1y} & r_{cy}+r_{bf3y} & r_{cy}+r_{bf5y}\\ r_{cx}+r_{bf1x} & r_{cx}+r_{bf3x} & r_{cx}+r_{bf5x} \end{array}\right]\cdot \left[\begin{array}{c} F_{g1z}\\ F_{g3z}\\ F_{g5z} \end{array}\right]=\left[\begin{array}{c} -m_{b}g\\ \tau_{ex}\\ \tau_{ey} \end{array}\right]$$

Then we can get the adjusted force directly by solving the inverse matrix without the usage of VTLSM.

## 5. Quasi-Incentre Stability Optimization

After buffer landing, the normal legs need to support the lander to finish the sampling task using the drill or spoon mounted on the body. If the fault does not occur, the ground projected point just locates on the center of the hexagon constructed by all leg footholds. In this case, the robot has the largest stability margin without adjustment demand for the center of mass. However, if several IDUs have failed, the old center of the hexagon does not coincide with the new center of the supporting polygon constructed by the remaining normal leg footholds. We should find a new center that maximizes the supporting stability margin.

### 5.1. Quasi-Incentre Definition

Here, we define the target center as a quasi-incentre that satisfies the following two/s: firstly, the sum value of distance ($d_{i}$) between this point and any sides of the supporting polygon is the maximum; secondly, all the distances $d_{i}$ should be as consistent as possible. Specifically, for regular polygons, the quasi-incentre is the center of the inscribed circle. As for the regular triangle, the quasi-incentre is just the incentre.

According to the definition of quasi-incentre, we can search the target point $q^{\;*}$ by the following function:

(17) $$\min\;f\left(q\right)=w_{1}\cdot \overset{n}{\underset{i=1}{\sum }}\frac{1}{d_{i}}+w_{2}\cdot \overset{n}{\underset{i=1}{\sum }}\frac{\left|d_{i}-d_{max}\right|}{d_{max}}$$

where n is the number of normal supporting legs, $d_{max}$ is the maximum distance between all $d_{i}$, $w_{1}$ and $w_{2}$ is the weight coefficients, $\overset{n}{\underset{i=1}{\sum }}\frac{1}{d_{i}}$ illustrates the first demand while $\overset{n}{\underset{i=1}{\sum }}\frac{\left|d_{i}-d_{max}\right|}{d_{max}}$ denotes the second demand.

### 5.2. Quasi-Incentre Searching

Particle Swarm Optimization (PSO) algorithm [32,33] is widely used in function optimization because it is simple to implement and has fewer adjusting parameters. In this algorithm, each particle possesses attributes of position and velocity. They are updated according to the following equation:

(18) $$v_{i}\left(k+1\right)=v_{i}\left(k\right)+c_{1}\cdot rand\left(\;\right)\cdot \left(Popt_{i}\left(k\right)-x_{i}\left(k\right)\right)+c_{2}\cdot rand\left(\;\right)\cdot \left(Wopt_{\;}\left(k\right)-x_{i}\left(k\right)\right)$$

(19) $$x_{i}\left(k+1\right)=x_{i}\left(k\right)+v_{i}\left(k+1\right)$$

where $x_{i}\left(k\right)$ and $x_{i}\left(k+1\right)$ are the position of the i-th particle at the k-th and (k + 1)-th iteration, $v_{i}\left(k\right)$ and $v_{i}\left(k+1\right)$ are the corresponding velocity, $rand\left(\;\right)$ is the random number between zero and one, $Popt_{i}\left(k\right)$ is the best position of i-th particle, $Wopt_{\;}\left(k\right)$ is the best position of the swarm, $c_{1}$ and $c_{2}$ are the learning factors. The actual particle velocity satisfies the following limits:

(20) $$v_{i}\left(k+1\right)=\left\{\begin{array}{c} \;\;\;v_{max},\;if\;v_{i}\left(k+1\right)\geq v_{max};\\ -v_{max},\;if\;v_{i}\left(k+1\right)\leq v_{max};\\ v_{i}\left(k+1\right),\;\;\;else. \end{array}\right.$$

where $v_{max}$ is the maximum of the searching velocity.

#### 5.2.1. Searching in V Configuration

**Figure 8:**
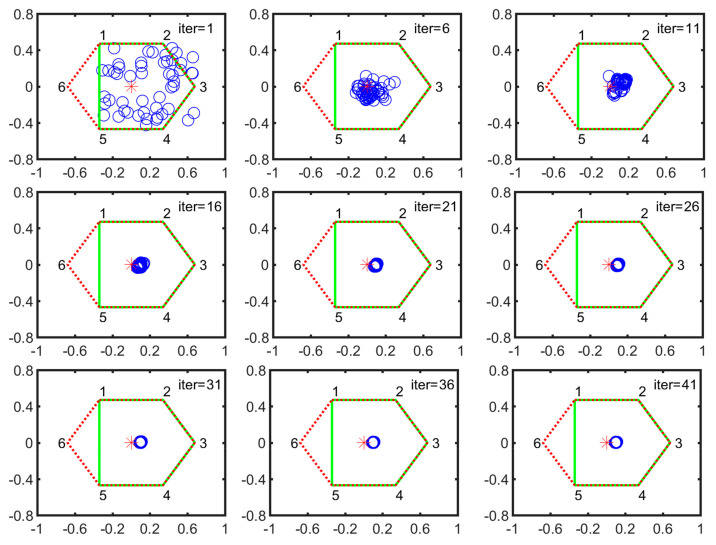
Particle evolution process in V-2 configuration (the horizontal axis denotes the forward/backward direction illustrated in Figure 2 while the vertical axis represents the leftward/rightward direction, respectively).

During five-legged landing, V-1 and V-2 are stable configurations. Here, we chose the V-2 configuration as the searching example. As shown in Figure 8, the numbers 1, 2, 3, 4, 5 and 6 denote the foothold positions of normal landing. All the particle positions at k-th iteration are expressed by the blue circles. The red star is the center of the hexagon that is constructed by all leg footholds and represented by the red dotted line. The new supporting polygon constructed by normal legs is expressed by the solid green line. Each subgraph is arranged in an increment of 5 iterations. At the first iteration, all the particles are scattered in the supporting plane. After 21 iterations, they are quickly concentrated into a small circular area. Then the particles slowly converge to the optimal position.

**Figure 9:**
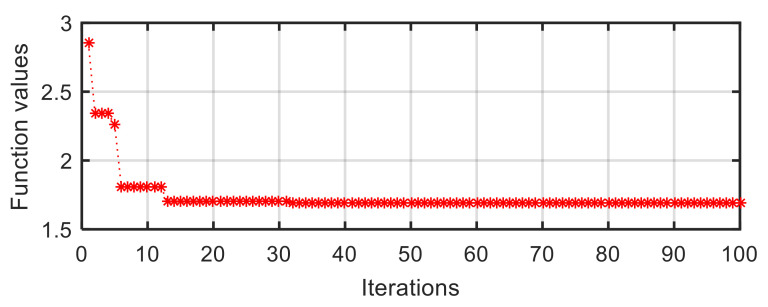
Target function value vs. iterations in V-2 configuration.

A similar process of particle convergence can also be found in the curve of target function value vs. iterations in V-2 configuration (Figure 9). At the first iteration, the function value is a larger number of 2.856. After a quick descent, it becomes a relatively stable value at the thirteenth iteration. Lastly, the value converges to a stable value of 1.692.

#### 5.2.2. Searching in IV Configuration

**Figure 10:**
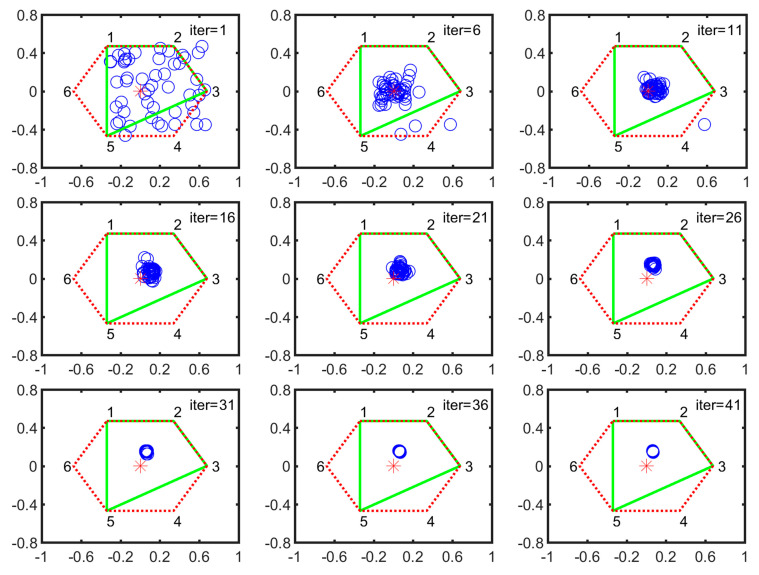
Particle evolution process in IV-2 configuration (the definition of vertical or horizontal axis is same as the one in Figure 8).

During four-legged landing, the stable configurations are IV-2, IV-3, IV-4 and IV-6. Here, we take the IV-2 configuration as an example to illustrate the converge process. Figure 10 shows the particle evolution process in IV-2 configuration. All particles are distributed in the supporting plane at the first iteration. After a quick evolution process, they concentrate on a small circle district, then converge to the optimal position with slow velocity.

**Figure 11:**
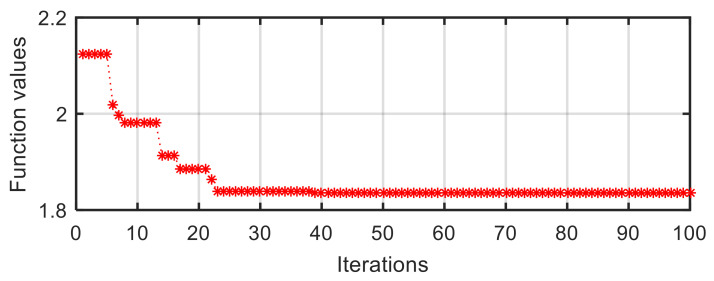
Target function value vs. iterations in IV-2 configuration.

At the same time, the converged process is detailly illustrated in the curve of target function value vs. iterations in IV-2 configuration (Figure 11). The initial target function value is 2.124 at the first iteration. After 23 iterations, it reduces to 1.839 rapidly. Eventually, the value converges to the stable value of 1.834 at a slow velocity.

#### 5.2.3. Searching in III Configuration

**Figure 12:**
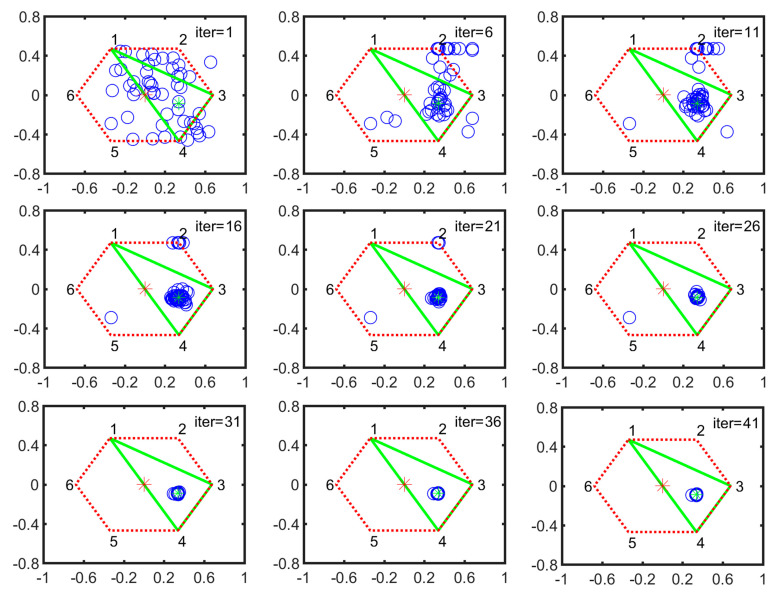
Particle evolution process in III-4 configuration (the definition of vertical or horizontal axis is same as the one in Figure 8 or Figure 10).

In a three-legged landing, the stable landing configuration is III-4. As is well-known, the triangle must exist in the center. In the III-4 configuration, its incentre coincides with the old center of the normal supporting hexagon and is set as the origin of the world coordinate frame. It is not necessary to search the quasi-incentre by the PSO algorithm. However, we can verify the target position obtained in the PSO algorithm by comparing it with the incentre. Here, we chose the III-3 configuration to check the searching result. The particle evolution process is shown in Figure 12. The green star is the theoretical incentre of the supporting triangle. After 31 iterations, the scattered particles concentrate to the incentre quickly and are limited in a small circle. Then they converge to the optimal position with a slow velocity.

**Figure 13:**
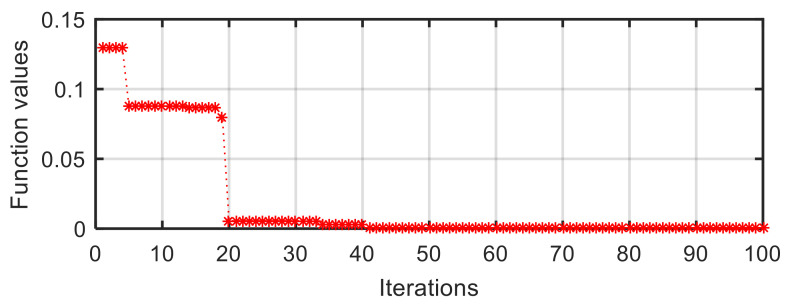
Target function value vs. iterations in III-4 configuration.

As shown in Figure 13, the curve of the target function value vs. iterations in the III-4 configuration can be divided into three terraces. The first terrace is the period from the first iteration to the fourth iteration; the second one is from the fifth iteration to the nineteenth iteration, while the third one is from the twentieth iteration to the final iteration. The corresponding function value reduces from initial 0.1302 to 0.0055, then to zero. The target function value of zero means that the distances between optimal position and any sides of the supporting triangle are equal, and the quasi-incentre is the incentre. Noticeably, we only use the second part in Equation (17) to achieve the searching in a supporting triangle while the weight coefficient of the first part is zero ($w_{1}=0$).

## 6. Experiments

In order to verify the fault-tolerant landing, we design a 5-Dof lunar gravity ground testing platform to provide the experiment scene. There were a series of experiments for stable configurations, including the five-legged, four-legged and three-legged soft-landing experiment conducted to verify the fault-tolerant landing capacity on the platform.

### 6.1. Experiment Platform

**Figure 14:**
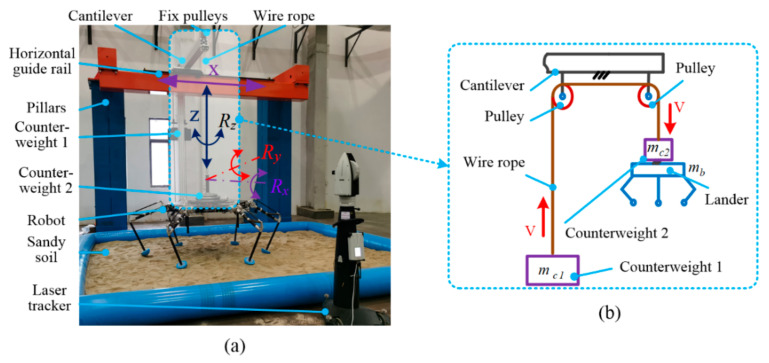
5-Dof lunar gravity ground testing platform. (**a**) components and Degrees of freedom; (**b**) components of counterweight system.

Figure 14a shows the detailed components, while Figure 14b expresses the construction of the counterweight system. The simulation capacity of the platform includes vertical movement z, horizontal x, and 3-Dof spatial rotation of $R_{x}$, $R_{y}$ and $R_{z}$. The design principle of counterweight is to make that the resultant force for the lander system in the vertical direction is equal to the one on the lunar surface by trickily dividing the load weight into two parts of counterweight one and counterweight two. In the experiments, we would simulate the soft-landing with a load of 140 kg on the Moon, which means the total mass ($m_{t}$) including lander and load is 180 kg, the counterweight one mass is 75 kg while the counterweight two mass is 65 kg.

### 6.2. Five-Legged Landing

**Figure 15:**
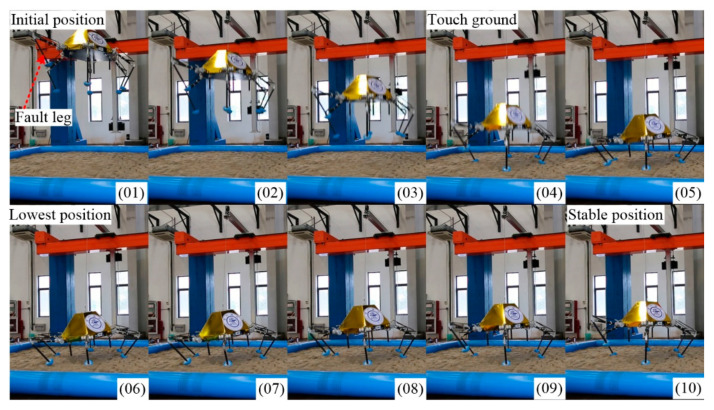
Keyframe snapshots in five-legged fault-tolerant landing.

As shown in Figure 15, the lander adopts V-2 configuration to finish soft-landing while the other configuration can bring out similar results. No. 01 is the initial position. No. 02 and No. 03 are declining in the air. No. 04 is the moment of full-touching with the ground. No. 05 is compressing to the lowest position (No. 06). After a damped vibration, the body reaches the stable position No. 10.

**Figure 16:**
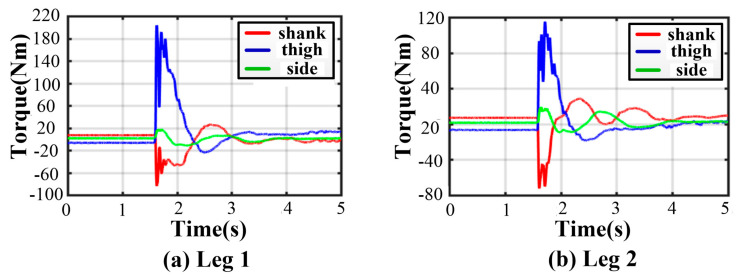
Joint torques in five-legged fault-tolerant landing.

**Figure 17:**
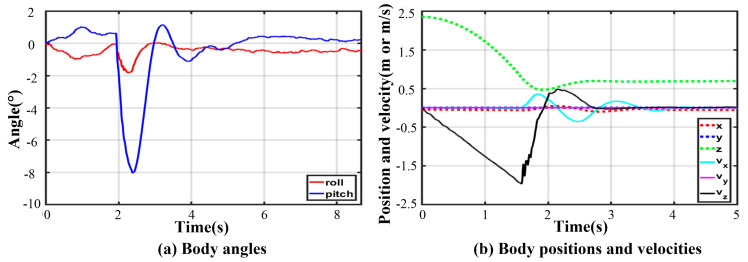
Body states in five-legged fault-tolerant landing.

Owing to the non-centrosymmetric pentagon constructed by the normal supporting leg, the difference of joint torques is great. As illustrated in Figure 16, the maximum of peak joint torques in each leg occurs in leg 1 while the minimum occurs in leg 2. The torque changes dramatically at the moment of touching the ground. The thigh peak torque is 203.7 Nm, while the shank peak torque is −84.14 Nm in leg 1. As for leg 2, the thigh peak torque is 115.1 Nm, while the shank peak torque is −72.47 Nm. The side joint torques are always small. After about a 2 s fluctuation, all joint torques reach a low, stable value. The curves of body angles are shown in Figure 17a. The fluctuation ranges are −1.8~0° and −7.98~1.13° for roll and pitch angles, respectively. The curves of position and velocity of the body are seen in Figure 17b. The lander touches the ground with a maximum velocity of −1.9 m/s in the z-direction. Next, the body continues to fall with a deceleration. After 0.36 s, the body velocity reduces to zero, and the body reaches the lowest position. Owing to the overturning force/torque caused by eccentricity, extra maximum velocities of 0.355 m/s and 0.04 m/s in the x and y direction are generated. Eventually, the body reaches a stable state after a damping vibration of 1.8 s.

### 6.3. Four-Legged Landing

**Figure 18:**
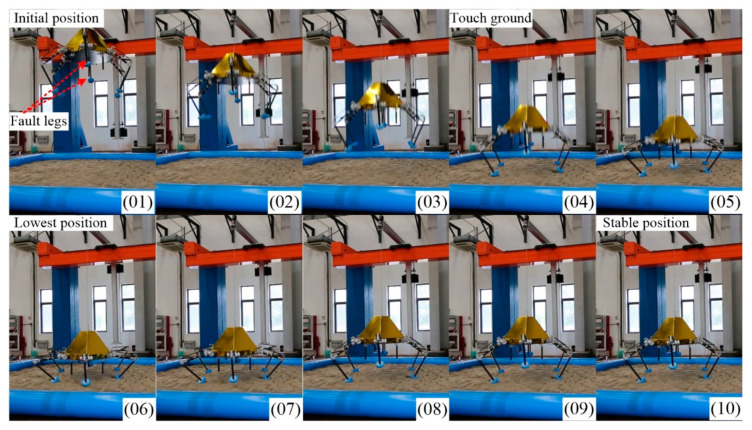
Keyframe snapshots in four-legged fault-tolerant landing.

Here, we chose the IV-6 configuration as the example to present the four-legged soft-landing. As shown in Figure 18, the buffer process in four-legged landing is similar to the one in five-legged landing. No. 01, No. 04, No. 06 and No. 10 are the initial position, touching ground moment, lowest position, and stable position, respectively.

**Figure 19:**
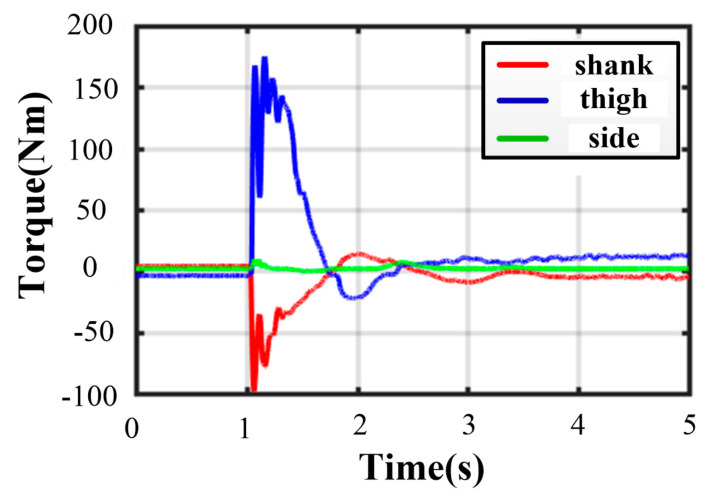
Joint torques in four-legged fault-tolerant landing.

**Figure 20:**
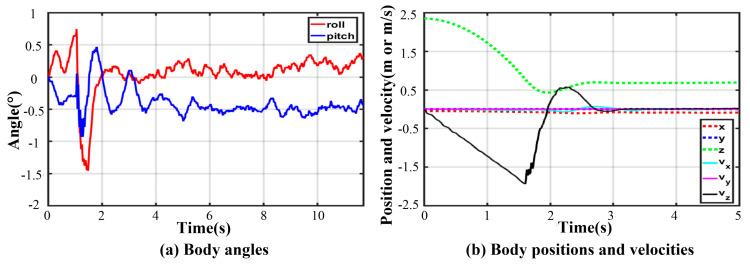
Body states in four-legged fault-tolerant landing.

Thanks to the centrosymmetric supporting rectangle, all curves of joint torque in each leg are basically the same. The maximum peak torque occurs in leg 4. As illustrated in Figure 19, the thigh peak torque is 175.2 Nm, while the shank peak torque is −98.39 Nm. At the moment of touching the ground, the torques of the thigh or shank and the angles of roll or pitch change greatly. As expressed in Figure 20, the maximum roll angle is −1.46° while the one of pitch angle is −0.93°. The touching ground velocity is the same as the one in a five-legged landing, but the fluctuation time is shorter and lasts about 1.6 s. The extra horizontal velocities in the x and y direction are almost zero because of the excellent symmetry of the supporting polygon.

### 6.4. Three-Legged Landing

**Figure 21:**
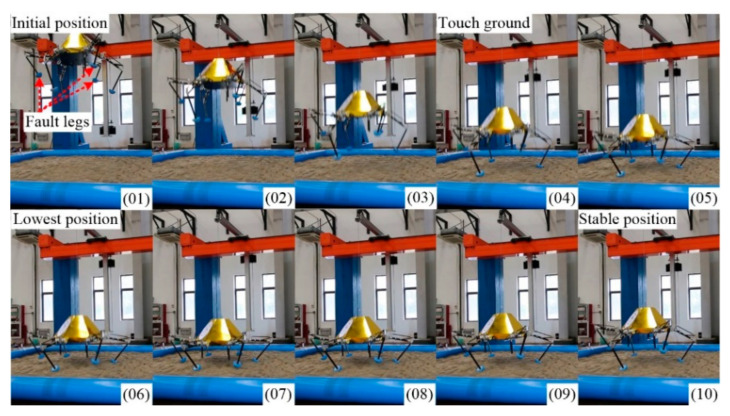
Keyframe snapshots in three-legged fault-tolerant landing.

**Figure 22:**
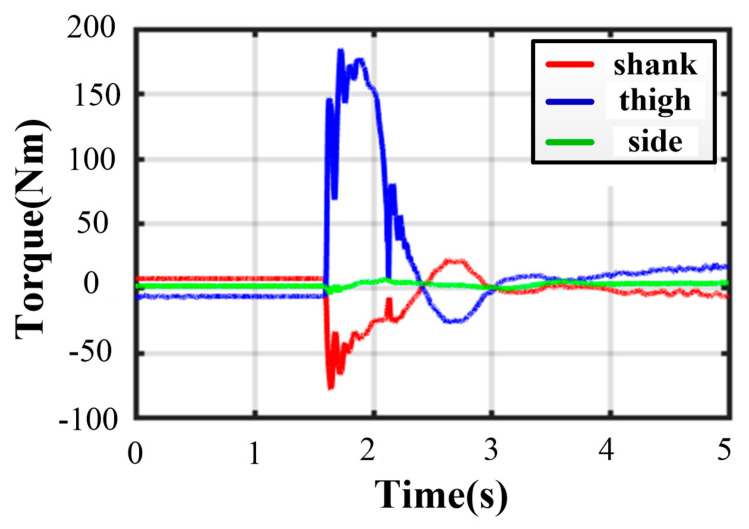
Joint torques in three-legged fault-tolerant landing.

**Figure 23:**
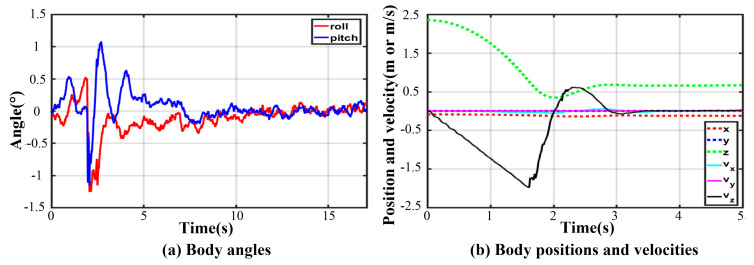
Body states in three-legged fault-tolerant landing.

As for three-legged landing, there is only one stable configuration denoted by III-4. Figure 21 shows the fluctuation process with damping vibration. The curves of joint torque in each leg are almost consistent, and the maximum peak torque occurs in leg 1. As illustrated in Figure 22, the thigh peak torque is 184.7 Nm, while the shank peak torque is −78.57 Nm. The fluctuation ranges of the angles of roll and pitch are −1.26~0.53° and −1.16~1.08° in Figure 23a, respectively. The maximum velocity in the z-direction is −1.9 m/s at the moment of touching the ground. The body velocity reduces to zero and reaches the lowest position after 0.416 s. Lastly, the body keeps a stable height by a damping vibration of about 2 s.

## 7. Discussion

As shown in Table 7, the soft-landing performances in different landing configurations with the same touch-ground conditions are obviously numerous. While the number of supporting legs has a great influence on landing performance, the spatial distribution of normal legs also plays an important role. In the four-legged and three-legged landing, all normal legs are evenly distributed, which generates almost no derivative velocity and results in a small angle derivation of roll and pitch (≈±1.5°). Thanks to more normal legs, the peak torque is smaller, and the damping vibration duration is shorter in four-legged landing than the ones in three-legged landing. As for five-legged landing, this case has the most supporting legs than three/four-legged landing, but its landing performance is not very great due to the terrible non-centrosymmetric distribution of normal legs. The indexes of peak torque and angle derivation in five-legged landing are worse than the ones in the centrosymmetric configuration, like four/three-legged landing. Furthermore, a derivative velocity of 0.355 m/s and 0.04 m/s in the x and y direction is separately generated due to the large angle derivation. Thanks to more supporting legs, the damping vibration duration in a five-legged landing is longer than the one in a four-legged landing, but it is still shorter than the one in a three-legged landing.

## 8. Conclusions

To execute the tasks of landing and roving simultaneously, a six-legged movable repetitive lander is designed and manufactured. Instead of absorbing the landing impact energy by irreversible deformation of aluminum honeycomb material in the current legged lander, a new electric IDU with high power, low weight and small volume is utilized to dissipate the energy actively by simulating the dynamic characters of spring and damper based on impedance control. The leg structure is still intact rather than permanent deformation, so the HexaMRL can perform repetitive exploration like the lander and rover. Fault-tolerant landing capacity is important for adapting this repetitive work mode. The main contributions are as follows:

(1) To achieve the soft-landing as far as possible with a failed IDU, we systematically analyze the classification and stability of landing configurations. Then the relationship between fault number and landing configuration is concluded by equation and listed by table.
(2) As for stable configuration, we have designed corresponding fault-tolerant landing algorithms to achieve buffer landing and further proposed a quasi-incentre stability optimization method to increase the stability margin during supported operations.
(3) A series of experiments including five-legged, four-legged and three-legged soft-landing with a vertical landing velocity of −1.9 m/s and a payload of 140 kg were conducted to verify the fault-tolerant landing capacity on the constructed 5-DoF lunar gravity ground testing platform by means of counterweight.

In future work, we will study the fault-tolerant walking capacity for HexaMRL to further improve the control theory under faults.

## Figures and Tables

**Table 1:** Fault-tolerant capacity of single leg.

Fault Number	Side	Thigh	Shank	Number	Symbol	*G_f_* Set	Fault Tolerance
1	LA	N	N	4	$E1_{1}$	$G_{F}^{II}\left(0,\;R_{\beta },0;T_{a},0,0\right)$	1
	LB	N	N		$E1_{2}$	$G_{F}^{II}\left(0,\;R_{\beta },0;T_{a},0,0\right)$	0
	N	L	N			$G_{F}^{II}\left(R_{\alpha },\;R_{\beta },0;0,0,0\right)$	0
	N	N	L			$G_{F}^{II}\left(R_{\alpha },\;R_{\beta },0;0,0,0\right)$	0
2	L	L	N	3	E2	$G_{F}^{II}\left(0,\;R_{\beta },0;0,0,0\right)$	0
	L	N	L			$G_{F}^{II}\left(0,\;R_{\beta },0;0,0,0\right)$	0
	N	L	L			$G_{F}^{II}\left(R_{\alpha },\;0,0;0,0,0\right)\;$	0
3	L	L	L	1	E3	$G_{F}^{I}\left(0,\;0,0;0,0,0\right)$	0

**Table 2:** Analysis table of landing configuration under fault combinations.

Type I	Number	Supporting Leg	Type II	Type I	Number	Supporting Leg	Type II
VI	$C_{6}^{6}=1$	1-2-3-4-5-6	VI-1	III	$C_{6}^{3}=20$	1-2-3	III-1
V	$C_{6}^{5}=6$	2-3-4-5-6	V-1			1-2-6	
		1-3-4-5-6				3-4-5	
		1-2-3-5-6				4-5-6	
		1-2-3-4-6				1-2-4	III-2
		1-2-4-5-6	V-2			1-2-5	
		1-2-3-4-5				1-4-5	
IV	$C_{6}^{4}=15$	3-4-5-6	IV-1			2-4-5	
		1-2-3-6				1-3-4	III-3
		2-4-5-6	IV-2			1-4-6	
		1-3-4-5				2-3-5	
		1-2-4-6				2-5-6	
		1-2-3-5				1-3-5	III-4
		2-3-5-6	IV-3			2-4-6	
		1-3-4-6				1-3-6	III-5
		2-3-4-6	IV-4			2-3-6	
		1-3-5-6				3-4-6	
		2-3-4-5	IV-5			3-5-6	
		1-4-5-6				2-3-4	III-6
		1-2-5-6				1-5-6	
		1-2-3-4					
		1-2-4-5	IV-6				

**Table 3:** Stability evaluation results of landing configurations.

Type I	Type II	Stability Radius *d*(m)	Supporting Area *S*(m^2^)	*SAI*	*SS*
VI	VI−1	0.5998	1.5233	1	S
V	V−1	0.3577	1.2640	0.4948	S
	V−2	0.4324	1.2803	0.6058	S
IV	IV−1	0	0.7617	0	CS
	IV−2	0.3577	1.021	0.3997	S
	IV−3	0.3577	1.0047	0.3933	S
	IV−4	0.4324	1.0047	0.4754	S
	IV−5	0	0.7617	0	CS
	IV−6	0.4324	1.0373	0.4908	S
III	III−1	−0.3577	0.2593	−0.1015	US
	III−2	0	0.5187	0	CS
	III−3	0	0.5023	0	CS
	III−4	0.3577	0.7617	0.2982	S
	III−5	0	0.5023	0	CS
	III−6	−0.4324	0.243	−0.1150	US

**Table 4:** Fault combination results.

N_m_	Solve Sets	Fault Legs Group	Type I	N_m_	Solve Sets	Fault Legs Group	Type I
1	{1,0,0}	$E1_{1}$	VI	4	{1,0,1}	$E1_{1}E3$	V
		$E1_{2}$	V			$E1_{2}E3$	IV
2	{0,1,0}	E2	V		{0,2,0}	E2E2	IV
	{2,0,0}	$E1_{1}E1_{1}$	VI		{2,1,0}	$E1_{1}E1_{1}E2$	V
		$E1_{1}E1_{2}$	V			$E1_{1}E1_{2}E2$	IV
		$E1_{2}E1_{2}$	IV			$E1_{2}E1_{2}E2$	III
3	{0,0,1}	E3	VI		{4,0,0}	$E1_{1}E1_{1}E1_{1}E1_{1}$	VI
	{1,1,0}	$E1_{1}E2$	V			$E1_{1}E1_{1}E1_{1}E1_{2}$	V
		$E1_{2}E2$	IV			$E1_{1}E1_{1}E1_{2}E1_{2}$	IV
	{3,0,0}	$E1_{1}E1_{1}E1_{1}$	VI			$E1_{1}E1_{2}E1_{2}E1_{2}$	III
		$E1_{1}E1_{1}E1_{2}$	V	6	{0,0,2}	E3E3	IV
		$E1_{1}E1_{2}E1_{2}$	IV		{1,1,1}	$E1_{1}E2E3$	IV
		$E1_{2}E1_{2}E1_{2}$	III			$E1_{2}E2E3$	III
5	{0,1,1}	E2E3	IV		{0,3,0}	E2E2E2	III
	{2,0,1}	$E1_{1}E1_{1}E3$	V		{3,0,1}	$E1_{1}E1_{1}E1_{1}E3$	V
		$E1_{1}E1_{2}E3$	IV			$E1_{1}E1_{1}E1_{2}E3$	IV
		$E1_{2}E1_{2}E3$	III			$E1_{1}E1_{2}E1_{2}E3$	III
	{1,2,0}	$E1_{1}E2E2$	IV		{2,2,0}	$E1_{1}E1_{1}E2E2$	IV
		$E1_{2}E2E2$	III			$E1_{1}E1_{2}E2E2$	III
	{3,1,0}	$E1_{1}E1_{1}E1_{1}E2$	V		{4,1,0}	$E1_{1}E1_{1}E1_{1}E1_{1}E2$	V
		$E1_{1}E1_{1}E1_{2}E2$	IV			$E1_{1}E1_{1}E1_{1}E1_{2}E2$	IV
		$E1_{1}E1_{2}E1_{2}E2$	III			$E1_{1}E1_{1}E1_{2}E1_{2}E2$	III
	{5,0,0}	$E1_{1}E1_{1}E1_{1}E1_{1}E1_{1}$	VI		{6,0,0}	$E1_{1}E1_{1}E1_{1}E1_{1}E1_{1}E1_{1}$	VI
		$E1_{1}E1_{1}E1_{1}E1_{1}E1_{2}$	V			$E1_{1}E1_{1}E1_{1}E1_{1}E1_{1}E1_{2}$	V
		$E1_{1}E1_{1}E1_{1}E1_{2}E1_{2}$	IV			$E1_{1}E1_{1}E1_{1}E1_{1}E1_{2}E1_{2}$	IV
		$E1_{1}E1_{1}E1_{2}E1_{2}E1_{2}$	III			$E1_{1}E1_{1}E1_{1}E1_{2}E1_{2}E1_{2}$	III

**Table 5:** Classification of virtual supporting triangle of stable configuration in five-legged soft-landing.

Type II	Valid Virtual Supporting Triangle	*N_v_*	Invalid Virtual Supporting Triangle	*N_i_*
V-1	2-3-5, 2-3-6, 2-4-5, 2-4-6, 2-5-6, 3-4-6, 3-5-6	7	2-3-4, 3-4-5, 4-5-6	3
V-2	1-2-4, 1-2-5, 1-3-4, 1-3-5, 1-4-5, 2-3-5, 2-4-5	7	1-2-3, 2-3-4, 3-4-5	3

**Table 6:** Classification of virtual supporting triangle of stable configuration in four-legged soft-landing.

Type II	Valid Virtual Supporting Triangle	*N_v_*	Invalid Virtual Supporting Triangle	*N_i_*
IV-2	2-4-5, 2-4-6, 2-5-6	3	4-5-6	1
IV-3	2-3-5, 2-3-6, 2-5-6, 3-5-6	4		0
IV-4	2-3-6, 2-4-6, 3-4-6	3	2-3-4	1
IV-6	1-2-4, 1-2-5, 1-4-5, 2-4-5	4		0

**Table 7:** The comparison of the key index in different fault-tolerant landings.

Index	Five-Legged Landing	Four-Legged Landing	Three-Legged Landing
Touch-ground velocity (m/s)	1.9	1.9	1.9
System mass (kg)	180	180	180
Thigh peak torque (Nm)	203.7	175.2	184.7
Shank peak torque (Nm)	−84.14	−98.39	−78.57
Roll angle derivation (°)	−1.8~0°	−1.46~0.74°	−1.26~0.53°
Pitch angle derivation (°)	−7.98~1.13°	−0.93~0.47°	−1.16~1.08°
Damping vibration duration (s)	1.8	1.6	2
Derivative *x* Velocity (m/s)	0.355	≈0	≈0
Derivative *y* Velocity (m/s)	0.04	≈0	≈0
